# Engaging Community and Healthcare Stakeholders in the Design of HIV Retesting Messages: Findings from Human-Centered Design Workshops in Kenya and Uganda

**DOI:** 10.64898/2026.04.28.26351157

**Published:** 2026-05-04

**Authors:** Megan A. Rabin, Alison M. Buttenheim, Kara Marson, Sabina Ogachi, Ronald Kisitu, James Ayieko, Jane Kabami, Moses R. Kamya, Shalin Desai, Kriti Chouhan, Gabriel Chamie, Harsha Thirumurthy

## Abstract

Frequent HIV testing, or “retesting,” the practice of regular HIV testing following a negative test result, among persons at high risk of HIV exposure is critical for initiating treatment early among newly infected persons and reducing the risk of HIV transmission. However, barriers to HIV retesting, such as fear of stigma, underestimating risk after a prior negative HIV test, and navigating the logistics of accessing an HIV test, have contributed to lower-than-desired retesting rates in Sub-Saharan Africa, where median time from infection to diagnosis is over 2.5 years. The Innovative Behavioral Intervention Strategies (IBIS) study aims to encourage re-testing by utilizing principles of behavioral economics and human-centered-design in a many-arm randomized trial (known as a “megatrial”) of avatar-delivered video-based messages and text messages to promote HIV retesting. In 2025, we conducted two-day focus groups in Kenya and Uganda to prototype the messages among community members and healthcare workers. An expert team engaged participants in various activities and discussions to elicit their feedback, where they reflected on factors such as local relevance, clarity, and visual appeal for each prototype. Key changes as a result of workshop feedback include standardized greetings for each arm, clearer language and refined translations, SMS language which protects participant privacy, and avatar updates for local acceptability, while maintaining core behavioral theory. The workshops generated important insights that shaped the final avatars, scripts, and messages encouraging HIV retesting which will be incorporated in the eventual trial. This study demonstrates the value of engaging end-users early in the intervention development process, and gives insight into the application of artificial intelligence (AI) to improve health behaviors in resource-limited settings.

## Full Text Availability

The license terms selected by the author(s) for this preprint version do not permit archiving in PMC. The full text is available from the preprint server.

